# *In-vitro* Activity of Avermectins against *Mycobacterium ulcerans*

**DOI:** 10.1371/journal.pntd.0003549

**Published:** 2015-03-05

**Authors:** Till F. Omansen, Jessica L. Porter, Paul D. R. Johnson, Tjip S. van der Werf, Ymkje Stienstra, Timothy P. Stinear

**Affiliations:** 1 Department of Microbiology and Immunology, The Peter Doherty Institute for Infection and Immunity, University of Melbourne, Parkville, Victoria, Australia; 2 University of Groningen, University Medical Center Groningen, Department of Internal Medicine/Infectious Diseases, Groningen, The Netherlands; 3 Austin Centre for Infection Research (ACIR), Infectious Diseases Department, Austin Health, Heidelberg, Victoria, Australia; 4 Department of Medicine, University of Melbourne, Heidelberg, Victoria, Australia; 5 University of Groningen, University Medical Center Groningen, Department of Pulmonary Diseases and Tuberculosis, Groningen, The Netherlands; Fondation Raoul Follereau, FRANCE

## Abstract

*Mycobacterium ulcerans* causes Buruli ulcer (BU), a debilitating infection of subcutaneous tissue. There is a WHO-recommended antibiotic treatment requiring an 8-week course of streptomycin and rifampicin. This regime has revolutionized the treatment of BU but there are problems that include reliance on daily streptomycin injections and side effects such as ototoxicity. Trials of all-oral treatments for BU show promise but additional drug combinations that make BU treatment safer and shorter would be welcome. Following on from reports that avermectins have activity against *Mycobacterium tuberculosis*, we tested the *in-vitro* efficacy of ivermectin and moxidectin on *M. ulcerans*. We observed minimum inhibitory concentrations of 4–8 μg/ml and time-kill assays using wild type and bioluminescent *M. ulcerans* showed a significant dose-dependent reduction in *M. ulcerans* viability over 8-weeks. A synergistic killing-effect with rifampicin was also observed. Avermectins are well tolerated, widely available and inexpensive. Based on our in vitro findings we suggest that avermectins should be further evaluated for the treatment of BU.

## Author Summary

Neglected tropical diseases such as Buruli ulcer predominantly afflict the poorest populations in the world and reduce quality of life. Buruli ulcer is a necrotising infection that destroys the skin and soft tissue, frequently presenting as nodules or open ulcers. Buruli ulcer is treated with antibiotics and sometimes surgery. Unfortunately the antibiotic treatment can have toxic side effects, such as hearing loss. Also, patients must either be hospitalized or report daily to a treatment centre to get their medicine as the treatment is delivered by injection.

In laboratory experiments we tested the susceptibility of *Mycobacterium ulcerans*, which causes Buruli ulcer, to avermectins. Avermectins are drugs that are used to treat common parasite and worm infections, such as river blindness. These drugs are inexpensive, have few side effects and are widely available. Our findings show that two avermectins called ivermectin and moxidectin inhibit the growth and also kill *Mycobacterium ulcerans* strains from both Africa and Australia. If their efficacy and safety also can be proven in animal and human studies, these drugs will provide an inexpensive addition to the current treatment of Buruli ulcer.

## Introduction

Buruli ulcer (BU) is a neglected tropical disease that presents as skin nodules, plaques or oedematous lesions that can progress to open ulcers [1]. BU is caused by infection with *Mycobacterium ulcerans*, a mycobacterium that is related to the causative agents of tuberculosis and leprosy [2]. Most BU patients are children under the age of 15 [3]. The mode of transmission of the disease is not well understood. Superstitious beliefs predominate in rural West Africa, resulting in delayed treatment seeking and stigmatization of patients [4,5]. Mortality associated with BU is low nevertheless morbidity is high. Extensive ulcers frequently lead to lifelong physical disability [6,7].

No vaccine is available against Buruli ulcer and management focuses on early case detection and treatment with surgery and antibiotics [8]. Previously, Buruli ulcer was treated with surgical excision only, but since 2004, an 8-week course of rifampicin and streptomycin is the standard treatment [9–11].

In case patients report early with limited lesions, the 8-week course of rifampicin and streptomycin delivers a good quality of life at long-term follow up [12]. However, the median time to heal is still 18 to 30 weeks, depending on the size of the lesion [10].

Patients presenting late to health care facilities have a much poorer prognosis and many suffer from functional limitations due to the disease [6,7].

There is also the issue that daily injections with streptomycin are impractical and can have serious side effects, such as ototoxicity [13]. It has been shown that all-oral treatment with rifampicin and a macrolide or quinolone results in high cure rates [14]. Shorter duration of antibiotic courses and more safe treatment regimes that reduce the time to healing are desirable for Buruli ulcer.

Avermectins are macrolides that are used to treat helminth-infection (such as strongyloidiasis or onchocerciasis) and parasitic infection (scabies) in humans and in animals. These orally administered drugs are well tolerated and available worldwide. Ivermectin is on the essential drugs list of the World Health Organization. A recent report showed that avermectins, including ivermectin, moxidectin and selamectin, inhibit the growth of different *M*. *tuberculosis* strains *in-vitro* at concentrations of 2 to 8 μg/ml [15]. Motivated by these findings, we tested if avermectins also inhibit and kill *M*. *ulcerans*.

## Materials and Methods

### Bacterial strains

Two different *M*. *ulcerans* clinical isolates were used, JKD8049 isolated from a patient in Victoria, Australia in 2004 and 1117–13, a 2013 clinical isolate from Benin. For time-kill assays (see below), *M*. *ulcerans* JKD8049 containing a bioluminescent reporter plasmid pMV306 hsp16+luxG13 [16] was employed. The *Mycobacterium marinum* ‘M’ strain was also used. Mycobacteria were grown at 30°C in 7H9 Middlebrook broth supplemented with OADC (Becton Dickinson, Sparks, MD, USA). *M*. *ulcerans* JKD8049 harbouring pMV306 hsp16+luxG13 was grown in the presence of 25 μg/ml kanamycin.

### Minimum inhibitory concentration (MIC) testing

Bacteria in mid-exponential growth phase were used for MIC testing. They were prepared to 0.5 Macfarlane standard and diluted 1:5 in PBS. A 500 μl volume of this preparation was used to inoculate duplicate BBL™ Mycobacteria Growth Indicator Tubes (MGITs) supplemented with 0.5 ml OADC (Becton Dickinson, Sparks, MD, USA) that contained doubling dilutions of moxidectin, ivermectin or rifampicin (Sigma-Aldrich, St. Louis, MO, USA.). The tubes were incubated at 30°C and assessed daily for fluorescence, with a long-wave UV-A lamp (Wood’s lamp) for 21 days. The tube with the lowest drug-concentration displaying no growth after this period was considered as containing the inhibitory concentration. Solvent only and rifampicin 0.1 μg/ml were used controls.

### Time-kill assays

Ten millilitre aliquots of mid-exponential growth phase *M*. *ulcerans* JKD8049 were transferred in duplicate to sterile 25cm^2^ tissue culture flasks containing 0, 8 and 20 μg/ml ivermectin. The aim was to obtain a *M*. *ulcerans* concentration of at least 10^6 CFU/ml in each flask. Each week, 50 μl of culture was sampled from each flask to assess viable bacteria remaining by CFU counting. Ten-fold dilutions of the sub-samples were prepared in PBS and a 3 μl aliquot of each dilution was spot plated in quintuplicate onto Middlebrook 7H10 agar containing 10% OADC. After 8 weeks of incubation at 30°C plates were examined for growth. The growth/no growth scores of the five technical replicates were used to calculate the most probable number estimate of CFU per ml. Data was analyzed using GraphPad Prism v5.0d.

### Bioluminescent kill curves

Three to five replicates of 200 μl aliquots of bioluminescent *M*. *ulcerans* JKD8049 in mid-exponential growth were transferred into a white 96-well plate and antibiotics were added. The plate was placed into a FLUOstar Omega plate reader (BMG LABTECH GmBH, Ortenberg Germany). Light emission was read every 300 s via the top optic with the gain set at 3600 and plate temperature at 30°C. Before each reading, plates were shaken at 100 rpm for 10 s in double orbital mode. The results were recorded using Omega v3.00 R2 and analyzed using Mars v3.01 R2 and GraphPad Prism v5.0d.

## Results

### *M*. *ulcerans* growth is inhibited by avermectins

*M*. *ulcerans* JKD8049, *M*. *ulcerans* 1117–13 and *M*. *marinum* were grown in MGIT tubes in the presence of increasing concentrations of the ivermectin and moxidectin. At three weeks, no fluorescence was observed at 8 μg/ml of ivermectin for *M*. *ulcerans* JKD8049 and at 4 μg/ml for *M*. *ulcerans* 1117–13 (Table 1). Moxidectin inhibited the growth of *M*. *ulcerans* JKD8049 at 4 μg/ml. The MIC for *M*. *marinum* was 32 μg/ml for ivermectin and above 64 μg/ml for moxidectin (Table 1). These data show that *M*. *ulcerans* but not *M*. *marinum* is susceptible to avermectins. Growth of all bacteria was observed in the control tubes containing only the solvent and no growth was observed in the tubes containing 0.1 μl/ml rifampicin.

**Table 1:** Minimal inhibitory concentration (MIC) of avermectins compared with rifampicin.

	MIC (μg/ml) ^1^		
	*M*. *ulcerans* JKD8049	*M*. *ulcerans* 1117–13	*M*. *marinum M*
Ivermectin	8	4	32
Moxidectin	4	nt^2^	>64
Rifampicin	0.1	0.1	0.1

^1^Minimum concentration to prevent MGIT fluorescence at 21 days;

^2^ not tested

### *M*. *ulcerans* killing by avermectins

**Fig 1:**
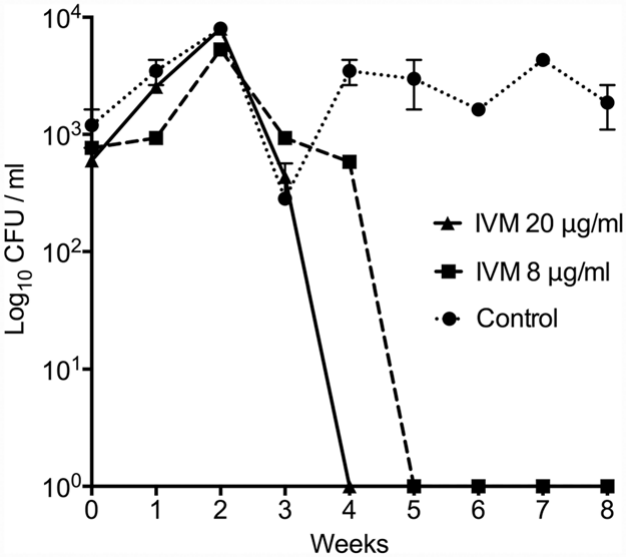
Time-kill assay showing the effect of ivermectin against *M*. *ulcerans* over 8-weeks. *M*. *ulcerans* was grown in 25cm^2^ culture flasks in the presence of 0, 8 and 20μg/ml ivermectin. Ethanol (solvent for ivermectin) at the concentration in the IVM-20 dose was used in the no-drug control. Weekly, ten-fold dilutions of each culture were plated onto 7H10 agar, using a 3-μl spot-dilution method, with five replicates per dilution. The plates were examined for growth after incubation for 8-weeks at 30°C, and colony forming units per ml calculated. The mean and range for duplicate biological experiments are shown.

Time-kill assays were then performed to assess if avermectins not only inhibit *M*. *ulcerans* growth but also kill the bacterium. Based on the MIC results above, a low and high dose of ivermectin was tested and *M*. *ulcerans* JKD8049 was exposed to either 8 μg/ml or 20 μg/ml ivermectin for eight weeks. A dose-dependent killing effect was observed with no CFU detected from week-4 onwards at 20 μg/ml and week-5 onwards for 8 μg/ml (Fig 1).

**Fig 2:**
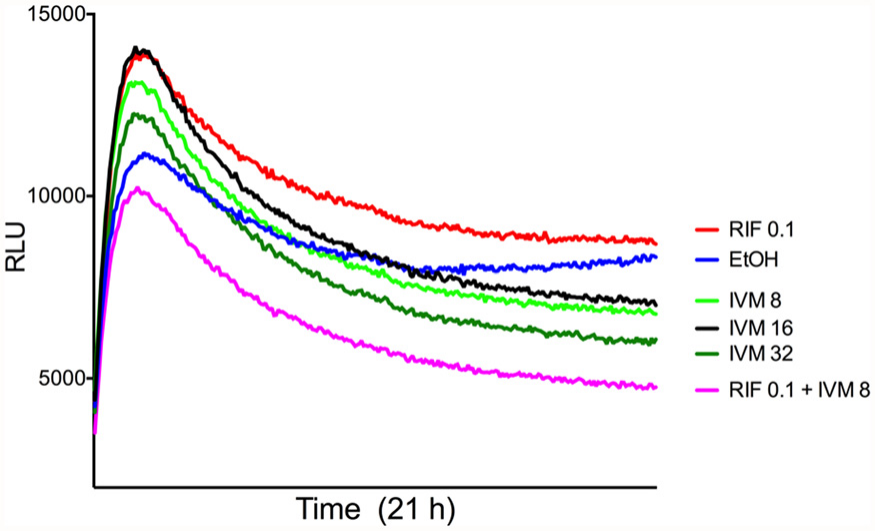
Dose-dependent change in relative light units (RLU) over 21 hours for bioluminescent *M*. *ulcerans* exposed to different concentrations and combinations of ivermectin and rifampicin. Ethanol (solvent for ivermectin) at the concentration in the IVM-32 dose was used in the no-drug control (EtOH). Depicted is the mean of at least technical triplicates for measurements made every 300 seconds over 21 hours.

Bioluminescence is an ATP-dependent process and it is therefore an excellent dynamic reporter of cellular metabolic activity [17]. A time-kill experiment was thus performed using bioluminescent *M*. *ulcerans*. Although the time frame of this experiment was quite short (21 hours), continuous monitoring over that period again showed a dose-dependent impact of ivermectin on bacterial viability (Fig 2). The reduction in bioluminescence at 21 hours was higher in ivermectin at all concentrations tested (8, 16 and 32 μg/ml) compared to the rifampicin positive control (Fig 2). Interestingly, the greatest impact on bacterial viability was observed in the presence of a combined dose of 8 μg/ml ivermectin and 0.1 μg/ml rifampicin (Fig 2).

## Discussion

The current treatment regimen of 8-weeks streptomycin and rifampicin for Buruli ulcer is highly effective but also problematic and promising alternative all-oral treatments are sought [13,14]. Here we have shown that ivermectin and moxidectin were able to inhibit the growth of different *M*. *ulcerans* strains at 4–8 μg/ml. These findings are comparable to previous research where MICs of 1–8 μg/ml against *M*. *tuberculosis* with ivermectin, selamectin, moxidectin or doramectin were reported [15].

*M*. *marinum*, despite its close relationship to *M*. *ulcerans*, showed low susceptibility to both ivermectin and moxidectin. We speculate that this difference may be explained at least in part by the abundance of intact transporters and efflux systems in *M*. *marinum* and a corresponding scarcity of the equivalent systems in *M*. *ulcerans* [18,19].

The 8-week time-kill experiment (Fig 1) showed that ivermectin at concentrations of 8 μg/ml and above has a likely bactericidal effect on *M*. *ulcerans*. Future experiments will test lower drug concentrations and use higher starting doses of bacteria. The long doubling time of *M*. *ulcerans* (>48h) complicates laboratory-testing methods using traditional culture and colony counting approaches. Here we used bioluminescence as a rapid read-out of cell viability and found it a useful technique for examining these slow-growing mycobacteria (Fig 2). Given the MIC findings (Table 1), we were surprised to observe in the bioluminescent time-kill experiments that ivermectin performed better than rifampicin at their given MIC concentrations. Further investigation of this difference is warranted, probably by conducting the experiments over an extended time period and beyond the 21 hours we were able to achieve here. A combination of 0.1 μg/ml rifampicin and 8 μg/ml ivermectin showed a synergistic killing effect. Clinically, rifampicin may however decrease the serum concentration of ivermectin by the induction of P-glycoprotein/ABCB1 in humans [20]. The extent to which this might be the case is not known and requires further testing.

In the context of avermectins to treat *M*. *tuberculosis* infections, it has been argued that MICs of 1–8 μg/ml are not achievable in humans [21]. Avermectins are mostly administered in much lower doses and in single-doses for the treatment of helminth infection. A maximum plasma concentration of 54.4 ng/ml was observed in healthy volunteers receiving 150 μg/kg ivermectin [22].

There are few public data on the safety of higher doses or prolonged courses of avermectins in humans and it unclear at what doses and over what time avermectins would have to be given to Buruli ulcer patients. We would argue that obtaining avermectin plasma concentrations at concentrations that approach MICs derived from in vitro experiments might not be needed for clinical efficacy. In our bioluminescent time-kill assay, 8 μg/ml ivermectin was superior to 0.1 μg/ml rifampicin. It may be that the treatment duration for BU can be reduced and that complete eradication of the microbe is not needed. Interference with the *M*. *ulcerans* mycolactone toxin synthesis machinery at concentrations that are sub-inhibitory for growth might be sufficient to permit host immunity to then clear the infection.

### Conclusions

The avermectins ivermectin and moxidectin inhibited growth of *M*. *ulcerans* at 4–8μg/ml and showed dose-dependent killing in culture-based and bioluminescence assays. The avermectins are inexpensive and already widely distributed in West Africa through their use to treat river blindness. Thus, there may be a chance to repurpose a well-tolerated drug for the treatment of mycobacterial infections, bypassing the long and expensive pipeline for discovery of new antimicrobials. We suggest that avermectins should be further investigated for the treatment of *M*. *ulcerans*, possibly in combination with other antibiotics, such fluoroquinolones.
